# An exploration of women's experience of taking part in a randomized controlled trial of a diagnostic test during pregnancy: A qualitative study

**DOI:** 10.1111/hex.12969

**Published:** 2019-10-02

**Authors:** Deirdre Hayes‐Ryan, Sarah Meaney, Caroline Nolan, Keelin O’Donoghue

**Affiliations:** ^1^ The Irish Centre for Fetal and Neonatal Translational Research (INFANT), University College Cork Cork Ireland; ^2^ Cork University Maternity Hospital (CUMH) Cork Ireland; ^3^ National Perinatal Epidemiology Centre University College Cork Ireland

**Keywords:** diagnostic test, preeclampsia, pregnancy, qualitative, randomized controlled trial, research

## Abstract

**Objective:**

To explore pregnant women's views of participation in a clinical research trial while pregnant.

**Design:**

Prospective nested qualitative cohort study embedded within a national, multi‐site randomized controlled trial of a diagnostic test for preeclampsia: Placental Growth Factor. One‐to‐one in‐depth semi‐structured interviews were undertaken with 19 women who had recently participated in the trial at a single recruiting site. The interviews were conducted in private, recorded digitally and transcribed verbatim.

**Setting:**

Single tertiary maternity hospital currently recruiting eligible women onto an on‐going randomized controlled trial (NCT 02881073).

**Participants:**

Women who had participated in the PARROT Ireland randomized controlled trial during their recent pregnancy.

**Methods:**

Thematic analysis was utilized. Each line of the transcribed interviews was coded into a category by two researchers. The resultant categories were reviewed, and those with similarities were pooled allowing the development of themes.

**Main Outcome Measures:**

Women's opinions and experience of participation in a randomized controlled trial of an interventional diagnostic test during their pregnancy.

**Results:**

Four major themes were identified as follows: (a) Understanding of preeclampsia, (b) Motivators for clinical trial participation, (c) Barriers to decision making and (d) Influence of PARROT Ireland on pregnancy experience.

**Conclusions:**

Women are generally interested and positively inclined to participate in research during pregnancy. The potential of risk is an important consideration for eligible pregnant woman. Information and support by both researchers and clinicians are paramount in aiding women's understanding of a research trial.

## 1 INTRODUCTION

A randomized controlled trial (RCT) is regarded as the gold standard when testing efficacy of any new drug, intervention or diagnostic test.^1^, ^2^ The use of drugs such as thalidomide and diethylstilbestrol in pregnant women has had long‐lasting repercussions, with women of childbearing age traditionally being excluded from clinical trials owing to safety concerns and fear of litigation.^3^, ^4^ Nevertheless, up to 74% of pregnant women take at least one mediation for chronic or acute conditions while the use of prescription medications by pregnant women has risen by more than 60% over the last three decades, with most of these drugs being used off‐label.^5^, ^6^ The paucity of evidence available on the use of medications in pregnancy means that some pregnant women may not be receiving optimal treatment, as clinicians are often unsure regarding correct dosage due to the physiological and metabolic changes that occur in pregnancy.^7^, ^8^ Further, lack of inclusion of women in clinical trials has resulted in a lack of evidence‐based care for pregnant women. Interventions such as cardiotocography and foetal fibronectin testing were integrated into clinical practice prior to robust evidence of their benefit.^9^ Once use of such clinical interventions is established within a system, withdrawal becomes challenging.^10^

In the last number of years, concerns have arisen over the ethics of actively excluding pregnant women from clinical trials.^11^, ^12^, ^13^, ^14^ In 1993, the Food and Drug Administration (FDA) lifted its ban on the testing of medicinal products in women and the National Institute for Health (NIH) legally endorsed the inclusion of pregnant women in trials.^15^, ^16^ Since the mid‐90s, there has been a concerted effort to ensure minorities, such as women and children, are represented in research in order to help guide scientific‐based practice for all societal groups.^17^, ^18^, ^19^ With the advent of perinatal research centres, each year more trials specific to pregnant women are developed, funded and conducted globally.^20^, ^21^ Literature is sparse in relation to women's willingness to take part in clinical research while pregnant.^22^, ^23^, ^24^ In addition, lack of experience in including pregnant women in trials may lead to poor trial design and hence recruitment difficulties.^25^, ^26^, ^27^ It is well documented that under‐recruitment is often an issue in RCTS, with a third not reaching target and over 50% requiring extensions.^28^ Given that we are involved in conducting an RCT of an interventional diagnostic test on a pregnant population, we determined we had a unique opportunity to explore pregnant women's views of participation in research, within a clinical trial. Our aim was to explore women's experience on being involved in a clinical trial, specifically a randomized controlled trial, while pregnant.

## 2 METHODS

This qualitative study was nested within a randomized controlled trial of a point‐of‐care diagnostic test (NCT 02881073) for pre‐term preeclampsia in Ireland. We first describe the PARROT Ireland RCT and then describe the nested qualitative study.

### 2.1 PARROT Ireland

PARROT Ireland was a multi‐site, national study recruiting women in the seven largest maternity units in Ireland, from 29th June 2017 until 26th April 2019. The trial aimed to examine whether the addition of point‐of‐care Placental Growth Factor (PlGF) testing to routine clinical care improved both maternal and neonatal outcomes for women with a pre‐term singleton pregnancy, and signs or symptoms of preeclampsia or placental dysfunction.^29^ If an eligible pregnant woman consented to participate, she was randomized to either control (routine care) or intervention (immediate additional PlGF testing) based on the current randomization of her hospital at the timepoint of her enrolment. As randomization was unblinded, both the participant and her clinical care team were aware of her allocation and her PlGF result if she was randomized to the intervention.

### 2.2 Nested qualitative study design

Participants at a single study site were given the opportunity to participate in the qualitative study. We employed a qualitative study design, using semi‐structured interviews, to explore women's views, experience and beliefs regarding pregnancy research. A semi‐structured topic guide was developed based on existing literature.^26^, ^30^, ^31^, ^32^, ^33^ Qualitative research has been utilized for many years to provide insight into problems, help develop hypotheses and to gain an understanding of underlying reasons, opinions and motivations.^34^ Interviews rather than surveys were employed as they facilitated a relationship of trust to be established between the researcher and participant.^35^ One‐to‐one interviews allowed for an environment where each participant was able to express themselves more openly than perhaps a focus group may have allowed.^36^

### 2.3 Recruitment

Purposive sampling of women who had recently completed the PARROT RCT was employed to ensure each arm of the trial was represented. Due to the stepped wedge design of the trial, the interval from recruitment until the interview was longer for those recruited in the control than the intervention. These women had previously consented to be approached about further trial‐related research. Each received a patient information leaflet and invitation to participate by post. Women who agreed to participate attended to the maternity hospital in‐person (n = 16) or were interviewed by telephone (n = 3) if in‐person attendance was not feasible. Informed written consent was obtained, and the interview process took approximately 30 minutes. Interviews were conducted over a three‐month period (September 2018‐December 2018) by DHR until data saturation was reached. At the time of interview, the PARROT Ireland trial was on‐going so results were not yet available to participants or researchers. Eighteen of the nineteen interviews were recorded digitally and transcribed verbatim. The one woman that did not wish to be digitally recorded gave consent for note taking by the researcher throughout the interview. These notes were used to inform the analysis.

### 2.4 Analysis

Interview transcripts were thematically analysed by DHR and SM.^37^, ^38^ In the initial analysis, each line of the transcript was coded into categories. The categories were then reviewed and refined, and themes were developed. These key themes were then presented and agreed upon by the entire research team.

## 3 RESULTS

Nineteen women were interviewed, ten of whom had been randomized to the control arm and nine who had been randomized to the intervention arm of the trial. Time from completion of study ranged from 6 to 15 months at the time of the interview. All women interviewed were Caucasian, age ranged from 24 to 42 years, and 52% were nulliparous prior to their recent pregnancy. Four themes were identified as follows: (a) Understanding of preeclampsia, (b) Motivators for clinical trial participation, (c) Barriers to decision making and (d) Influence of PARROT Ireland on pregnancy experience. Direct quotations from the women, presented in‐text, are used to illustrate these themes.

### 3.1 Understanding of preeclampsia

Women were recruited to the PARROT Ireland trial on the basis that they were exhibiting signs or symptoms of pre‐term preeclampsia in their pregnancy. Some women were diagnosed with preeclampsia. They described the significant impact this had on their pregnancy, emphasizing how it took over their lives leaving them with a lack of control and autonomy.

…suddenly I was in hospital and I didn't leave for a month. I had loads of planning to do and my mat leave was happening the next day and just small things that were kind of taken away in a sense, putting the nappies in where I wanted to put them and the vests and washing them and all those little preparation stuff that I was kind of looking forward to P8

Most of those interviewed reported only having vaguely heard of the condition prior to their pregnancy with friends or social media being the main source of their knowledge. As most of the participants were nulliparous women at the beginning of their pregnancies, these women did not feel that preeclampsia was of concern to them.

It didn’t really affect me, it wasn’t anything to do with me P3

Most of those interviewed were not aware of risk factors for preeclampsia and had misconceptions about who might develop preeclampsia. Women commonly stated that they were shocked when the possibility of preeclampsia arose later in their pregnancy.

Like I thought it was something kind of third world people got P7

I would have heard of it, but I wouldn’t have known much about it P15

Before being pregnant I don’t think… maybe I had heard the word pre‐eclampsia, but I definitely wouldn’t have known any specific information P17

Those who had been pregnant previously, especially those previously investigated for preeclampsia, had better knowledge of the risks, symptoms and potential consequences of the condition.

I definitely thought that you were overweight and unfit and like you'd brought it on yourself kind of thing P9

I was definitely more aware of it, because they had mentioned it’s a possibility or that it is more probable the second time round. So, I would have been aware of at least the symptoms of it and watching out more in the second pregnancy P17

When subsequently informed that they required investigation for preeclampsia, women were eager for simple, clear, concise information about the condition from a reliable source. Some felt they got the necessary information from their clinical care team. Others searched for the information themselves, usually using online sources, and often felt overwhelmed at the vast amount and varied quality of information available.

You don't know where to look regarding wanting to get correct information because there's so much now online P8

### 3.2 Motivators for participation in clinical research

Despite limited previous experience with participation in research, especially medical research, the women had extensive knowledge regarding research. All were convinced about the merit and importance of research, especially in pregnancy, for increasing knowledge of conditions and improving future clinical care.

I just think information is power and the numbers don't lie and if you have information, you can do something P9

I felt like whatever we could do it would be a benefit, so I was happy to participate to be honest P13

Almost all of those interviewed reported it was a straightforward decision to take part in PARROT Ireland, and that it did not require a lot of time for consideration nor involve discussion with others.

…as far as I was concerned it was my body and they didn’t really have a say P2

The study was quite easy‐going, you know, it's a blood test or no blood test and then a questionnaire, so that was no real decision on my part in that it was handy enough P13

The women reported altruism and the potential to help others in the future as a key motivator for participation. Many felt their contribution was essentially ‘paying it forward’ for the knowledge that could be garnered from their current pregnancies in order to benefit those in the future. A second key motivator to participate was a lack of burden associated with participation in the PARROT trial. Even though women reported they would be happy to give extra time or attend for extra appointments if required for research purposes, one of the main factors was how straightforward it was to take part in the PARROT trial.

other people being involved in research previously surely helped me when I was pregnant P3

the fact that it was so simple….you could say yes or no…and then you have a blood test or you didn’t and then there was no extra travel or filling out huge surveys or anything like that P17

The potential of participation facilitating an opportunity of an earlier diagnosis, or identification of a problem, also influenced women's decision to enrol. Women felt that by being part of the study they might know sooner than others if they developed a pregnancy complication. The demeanour of the PARROT researcher was also reported to be an important factor in their decision to take part. Women discussed the researcher's style of approaching eligible women remarking that the researcher was kind and friendly, made them feel at ease, explained the study in clear simple terms, all without rushing or pressurizing women in any way.

she couldn’t have been nicer, she really couldn’t…had she not been so sensitive and understanding I possibly wouldn’t have you know P5

It was completely up to me and I didn’t feel at all under pressure. I mean I could have just left it. I remember there was a window just next to me so I could have just left it on the window sill and you know, nothing would have ever come of it P17

Women reported being positively influenced to take part if they heard about the trial from their treating clinician. Most believed the trial would not have been suggested to them unless it was useful and would be beneficial to them. They also reported being more likely to take part if they recognized the name of one of the principal investigators (PI) of the study. The women discussed how these PIs are senior consultants who are well respected clinically and are highly sought after for private obstetric antenatal care locally.

I think that was less pressure in a sense, because you know when the researcher approached me directly, it’s kind of my responsibility “do you want to take part?” whereas when it comes from you know your consultant it’s an easier step to take then P5

### 3.3 Barriers to decision making

The main deterrent to participation in clinical research identified by the group was risk. The group reported being highly reluctant to take part in any research should they perceive it as being potentially harmful to the developing foetus. Taking a medication while pregnant, as part of a research trial, was an example of what women would consider risky. Most would be very reluctant to take part in such a trial and admitted it would require very careful consideration and discussion with their partner if they were approached in the future about such a research study.

I'd be very slow, I'd have to really think about it, purely because it's somebody else's life you're putting on the line, not just your own, it’s somebody else's future P7

The potential requirement for blood tests from the woman as part of a clinical study was not found to be a deterrent among the group; however, if blood sampling was required from the baby following delivery, this would be considered a deterrent. Any test that was invasive, and potentially would cause distress or pain to the baby, that was not required outside of a research setting would not be well received.

You don't want to be the guinea pig and you certainly don't want the baby to be the guinea pig either… you're like well can some people in some other countries sign up first and see what the outcome is. You always want somebody else to stick their toe in the water first P9

The clinical situation of the individual woman, at the time point when she is approached to participate in a trial, was flagged by those interviewed as important to consider. Many participants reported that if they had recently received sensitive or distressing information or were currently experiencing serious complications in their pregnancy; they may have been less willing to take part in the trial. The women interviewed mentioned that the language used by researchers when approaching eligible pregnant women is important to consider. Using complicated words and medical jargon could be frightening for some women and many stated it would sway them against participation.

Like I do think it is very dependent on you know the news that you have been given, like if you have been given very sensitive news, you might feel like, why should I be…. the guinea pig to help future cases P5

I think any pregnant woman would happily take part, I think the only time that someone might not want to do it is if they are facing a crisis, and they are in a bit of a fog and they can’t really think P2

I think the only thing that would turn people off is saying its medical research –that can be scary. Maybe, say its more for women’s health P4

### 3.4 Influence of PARROT on pregnancy experience

When approached for further interview, all those who agreed to take part reported recalling the trial and their agreement to take part. The name of the trial, PARROT, was found to be memorable. Most were uncertain as to why a trial concerning pregnant women and preeclampsia had used an acronym representing a bird. Some understood this was an abbreviation for a longer name. Nobody reported the name to have negative connotations, but some did mention that if it had mentioned a baby in the title, it may have been more inviting.

no I remember the name because I have a parrot! So, if it had been another name … but I remember this P6

It did cross my mind…”why parrot”? but I didn’t think too much about it really. I remembered it straight away so maybe the name was good P14

All enrolled participants in PARROT Ireland were asked to complete a five‐page paper Quality of Life questionnaire at the time point of their enrolment and again prior to their discharge from hospital postnatally. When asked about their thoughts on the questionnaire, most respondents had little memory of it and did not report it as being off‐putting or time‐consuming. Some commented that they were a welcome distraction while waiting in a busy antenatal clinic and allowed them time to reflect on their current self.

The researcher … she was dropping the follow‐up questionnaire, so she could know if I needed to get on to somebody P13

One thing I did like about it was it makes you think, it made me think about where I was at in the weeks after having my baby, it gives you time to think what level of anxiety am I now, so that was nice, that was a really good benefit of it P8

The majority of participants were aware of the concept of randomization in a research setting and recognized the importance of assigning participants to groups in order to be able to examine the outcomes when evaluating a new test, etc

I guess controls are important I suppose, to some extent you know, you need a certain amount of people to take part in order to get a certain level of statistics, that you need you know P10

While I would still like to be part of the group of people that get the test, for research to develop and everything they need people on the other side of it, so I suppose that I would accept the plan whatever it was P12

A distinct difference was expressed between those in the control and those in the intervention regarding the overall experience of the trial on their pregnancy. Those from the control group had a poorer recollection of what the purpose of the trial was and that a blood test was offered to half the cohort. The women recruited to the control arm felt that the trial did not impact on or influence their pregnancy in any way but were still happy to have taken part.

It was really personable, it actually wasn’t like we were just another case number. If anything it brought me on sense of…somethings going right. Like there was never anything bad to come out of the trial for me, like worst case scenario you got nothing, you were just like you were when you started. it was all beneficial in one form or another depending on what way you looked at it P2

I suppose, all I was thinking of was if it will help me or help other people I will. But to be honest information probably went a little over my head, I actually can’t tell you now what the Parrot Study is, and that ….through no fault of anybody, but I personally had so much going on P16

Those who were in the intervention arm had a better recollection of the purpose of the trial and overwhelmingly felt being enrolled onto the trial was beneficial to their pregnancy. They felt that knowledge of the PlGF result, whether it was normal or abnormal, was useful to the clinicians caring for them and positively influenced their pregnancy. They also felt they received extra care from their clinicians due to their involvement in the trial and having had the extra blood test performed.

I just felt that everyone was giving the best care and all of this research and all of this information was for my baby’s good so I thought it was a very positive thing P18

I thought it was very good, to be honest. It was hands‐on like, you know. I don’t remember ever a doctor ringing me like, so….I was happy with that. I think it put a rush on me being monitored, if I’m being honest, it was definitely beneficial P18

## 4 DISCUSSION

This qualitative study brings together insight into women's decision making regarding participation in an interventional clinical trial during pregnancy. We identified that pregnant women are aware of the importance of conducting research and are interested in taking part, provided participation does not put their unborn baby at any risk. We found there was limited background knowledge of preeclampsia among the group and women wanted information on this condition to be clear, concise and provided by a reliable source. In our study, those randomized to the intervention felt participation in the trial directly benefited their pregnancy, with the additional test providing valuable information on placental functioning and the perception of increased care from clinicians. On reviewing the literature, we identified limited numbers of previous studies examining women's experience of participation in a RCT while pregnant.^30^, ^31^, ^32^, ^33^, ^39^ These RCTs vary in terms of design and methodology, frequently involved administration of a medicinal product or a placebo. Given that our RCT employed a diagnostic test as the intervention, we identified a novel opportunity to gain insight and add new knowledge to this under‐examined area.

Participants of our nested study reported both altruism and the potential of personnel benefit to be key motivators in their enrolment in PARROT Ireland. The prospect of an additional blood test, with its potential of earlier identification and diagnosis of a clinical complication, was an incentive for our study. Others have similarly reported a sense of civic duty, the opportunity to help others and the possibility of an improved outcome for their baby to be driving forces behind participation.^30^, ^32^ A Brazilian group reported the main motivator to comprise access to free medicine and an opportunity to engage with health‐care providers.^31^ This highlights that in countries where health care during pregnancy is not publicly available, participation in clinical research may be the only option those with limited financial means have in order to access medical care. In such cases, governance of research trials must be closely regulated to ensure this vulnerable group are not exploited.

Respondents in our study reported being more likely to take part in the trial if it was mentioned to them by their treating clinician or a member of the medical team. Endorsement of the trial from medical personnel appears to validate a study for patients. Similarly a lack of interest or support from local clinical staff has been reported as a barrier to participation.^26^ Accurate knowledge by clinicians of on‐going trials in their unit and the vocalization of their support is crucial in promoting participation of pregnant women in future trials.

This nested study identified that the main barrier preventing participation of pregnant women in clinical research is the potential of causing harm to the baby. Others have also found pregnant women to be risk adverse, with apprehension and risk limitation being common barriers prohibiting participation.^26^, ^32^ Clinical trials require sponsorship, insurance and undergo rigorous review by national ethical committees prior to their commencement. On‐going clinical trials are vigilantly monitored by stakeholders, to ensure any trends in adverse events are quickly detected and can be acted upon with possible cessation of the trial if necessary.^11^, ^21^, ^40^ Changing a pregnant woman's perception of risk is key. Education, through the information and explanation provided by researchers, is paramount. Adequate training of researchers and clinicians, to maximize this skill set, should be prioritized for future studies.

This study revealed that the decision to take part in the PARROT Ireland trial was made independently by the pregnant woman herself, without any consultation with her partner, friends or family. Similar findings were reported in the QUOTE study,^33^ while in contrast the RIPE study 32 reported equal involvement of both the women and her partner in the decision to take part in an RCT. Both RCTs involved taking a medication while pregnant; however, in QUOTE women had preeclampsia when enrolled, whereas in RIPE healthy pregnant women were recruited. This finding highlights trials that involve taking a medication while pregnant, especially if recruiting healthy pregnant volunteers, likely require a longer time interval from first approach by researchers until signing consent, to facilitate shared decision making.

Women reported feeling well informed about the PARROT Ireland trial prior to signing the consent and later had a good understanding of the trial when interviewed. Respondents reported that the timing and setting of the researcher's approach were appropriate and the language used was understandable and unambiguous. In contrast, participants of both the MAGPIE^41^ and the ORACLE^42^ RCTs reported confusion when the trial was initially explained to them. They did not fully understand that randomization meant they might not receive the intervention and subsequently had limited knowledge and recall about the trials.^30^, ^33^ This difference may be attributable to the clinical situation of the women at the time of recruitment. For PARROT Ireland, eligible women were approached in a variety of clinical settings, antenatal clinics, wards, assessment units, all while undergoing routine assessments. Both the MAGPIE and ORACLE trials recruited women in Labour Ward/High Dependency Ward, either in pre‐term labour or close to indicated emergency delivery. Given the complexity of these clinical situations, it is plausible that women may feel overwhelmed and unable to clearly assimilate information provided about a research study. Designing future trials with recruitment focused in non‐emergent situations may provide a solution to this, and ensure patient vulnerability is not exploited. An alternative could be to employ the use of a delayed consent process for labour ward‐based trials.^43^ This approach has been employed in trials of critically ill patients, is well described and has been found to be acceptable to patients.^44^

The women in our study randomized to the control did not report negative experiences. Although they felt the trial had no direct impact on their pregnancy, they were still happy to have taken part. In contrast to our results, others have reported randomization to the control of an RCT perceived as being disadvantageous.^32^ A loss of equipoise on the subject under investigation may be one explanation or equally a familiarity of the intervention among the population. Prior to randomization in a study, if participants have strong favourable personal opinions on the product being investigated, it may lead to disappointment and disillusionment if they then are randomized to the control. This highlights the need for education of eligible participants by researchers on the purpose and importance of both arms of a RCT. It also highlights the impact background knowledge of the topic under review in the eligible population may have on their willingness to participate and needs to be considered by researchers when planning future studies.

The women we interviewed are likely to be highly motivated and interested in research as they not only took part in the trial during their pregnancy, but also agreed to participate in the qualitative study. Ideally, we would have interviewed those who declined to take part in the trial also, as this would have better elicited the barriers to research participation in pregnancy. However, as per Good Clinical Practice (GCP)^45^ and General Data Protection Regulations (GDPR)^46^ we did not retain any information on eligible women who were approached but declined to participate in the trial, thus contacting them for this study was not feasible. Strengths of our study include in‐person interviews, facilitating a more personal relationship between the researcher and participant as well as close proximity of interview to time of participation, which greatly aided participants recall. Uniquely, the PARROT Ireland RCT was not blinded; hence, even though the trial has not yet published, women subsequently interviewed were aware of their randomization and, for those in the intervention, knew their PlGF result.

Our findings correlate well with those from a recent systematic review examining the facilitators and barriers to pregnant women's participation in research.^39^ It reported altruism and the potential to contribute to science to rank highly as motivators to women's participation. The potential for personal benefit, through increased surveillance or earlier detection of medical conditions, was also a commonly reported motivator. Similar to our nested study, the systematic review reported pregnant women to be risk adverse with the potential requirement of taking a medication while pregnant a major barrier to participation. Unlike our study, the review reported personnel inconvenience as a barrier to participation among pregnant women. This difference may potentially be explained by the design of our trial, with no on‐going assessments or repeat attendance required it was well received by participants. Another difference between the two studies was an underlying distrust of researchers identified by the authors of the systematic review. This difference may possibly be explained by the demeanour and approach adopted by the research midwife of our study. Her candour and non‐pressurizing approach were frequently positively commented on by participants.

## 5 CONCLUSION

This study highlights that pregnant women are aware of the importance of research and are generally interested and positively inclined to participate. It identifies that the context, purpose and potential risk of any research are the most important considerations to an eligible pregnant woman. The approach and explanation adopted by both researchers and clinicians are paramount in aiding women's understanding of a research trial. This information may aid the design and conduct of future studies, thereby increasing their acceptability for pregnant women.

## CONFLICT OF INTEREST

The authors report no conflicts of interest.

## AUTHOR CONTRIBUTIONS

All authors contributed to the overall study design and specific methodologies. KOD and SM conceived and designed the study with DHR. DHR conducted the data collection with assistance from CN. DHR conducted the analysis with assistance from SM. DHR drafted the manuscript with assistance from SM and KOD. All authors have critically read, contributed with inputs and revisions and approved the final manuscript.

## ETHICAL APPROVAL

Ethical approval for the study was granted from the Cork Research Ethics Committee; a nationally recognized research ethics committee (CREC ECM 4 (t) 14/08/18).

## Data Availability

Due to the nature of the study, it is not feasible to anonymize the interview transcripts. Therefore, primary study data will not be made available publicly or to other researchers.
